# *catena*-Poly[[tetra­kis­(1*H*-pyrazole-κ*N* ^2^)copper(II)]-μ-hexa­fluoridosilicato-κ^2^ *F*:*F*′]

**DOI:** 10.1107/S1600536812009531

**Published:** 2012-03-14

**Authors:** Hui Li, Qiuping Han, Xiaochuan Chai, Jian Wang, Chenzhong Yao

**Affiliations:** a Department of Applied Chemistry, Yuncheng University, Yuncheng, Shanxi 044000, People’s Republic of China

## Abstract

In the title one-dimensional coordination polymer, [Cu(SiF_6_)(C_3_H_4_N_2_)_4_]_*n*_, the Cu^II^ atom is coordinated by two hexafluoridosilicate F atoms and four pyrazole N atoms in a distorted *trans*-CuF_2_N_4_ octa­hedral environment. The dihedral angle between the planes of the pyrazlole rings in the asymmetric unit is 74.4 (3)°. The hexa­fluoridosilicate dianion acts as a bridging ligand, connecting the Cu^II^ atoms into a [1-10] chain. The Cu and Si atoms lie on special positions with 2/*m* site symmetry. In the crystal, intra­chain N—H⋯F hydrogen bonds occur and weak C—H⋯F inter­actions link the chains.

## Related literature

For background to coordination polymers with nitro­gen-containing ligands, see: Li *et al.* (2011 ▶).
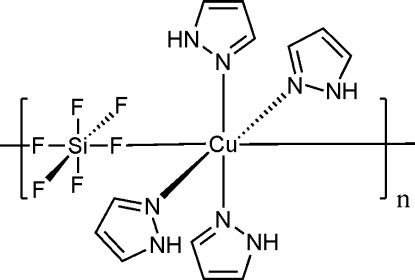


## Experimental

### 

#### Crystal data

[Cu(SiF_6_)(C_3_H_4_N_2_)_4_]
*M* *_r_* = 477.96
Monoclinic, 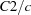

*a* = 10.617 (2) Å
*b* = 12.108 (2) Å
*c* = 14.652 (3) Å
β = 95.07 (3)°
*V* = 1876.2 (6) Å^3^
*Z* = 4
Mo *K*α radiation
μ = 1.30 mm^−1^
*T* = 293 K
0.25 × 0.22 × 0.20 mm

#### Data collection

Rigaku Mercury CCD diffractometer
Absorption correction: multi-scan (*CrystalClear*; Rigaku/MSC, 2005 ▶) *T*_min_ = 0.730, *T*_max_ = 0.771
7864 measured reflections
1665 independent reflections
1283 reflections with *I* > 2σ(*I*)
*R*_int_ = 0.079

#### Refinement

*R*[*F* ^2^ > 2σ(*F* ^2^)] = 0.059
*wR*(*F* ^2^) = 0.101
*S* = 1.16
1665 reflections
130 parameters
H-atom parameters constrained
Δρ_max_ = 0.44 e Å^−3^
Δρ_min_ = −0.31 e Å^−3^

### 

Data collection: *CrystalClear* (Rigaku/MSC, 2005 ▶); cell refinement: *CrystalClear*; data reduction: *CrystalClear*; program(s) used to solve structure: *SHELXS97* (Sheldrick, 2008 ▶); program(s) used to refine structure: *SHELXL97* (Sheldrick, 2008 ▶); molecular graphics: *SHELXTL* (Sheldrick, 2008 ▶); software used to prepare material for publication: *SHELXTL*.

## Supplementary Material

Crystal structure: contains datablock(s) I, global. DOI: 10.1107/S1600536812009531/hb6651sup1.cif

Structure factors: contains datablock(s) I. DOI: 10.1107/S1600536812009531/hb6651Isup2.hkl

Additional supplementary materials:  crystallographic information; 3D view; checkCIF report

## Figures and Tables

**Table 1:** Selected bond lengths (Å)

Cu1—N1	2.007 (4)
Cu1—N3	2.008 (3)
Cu1—F3	2.348 (2)
Si1—F1	1.679 (2)
Si1—F2	1.680 (3)
SI1—F3	1.695 (2)

**Table 2:** Hydrogen-bond geometry (Å, °)

*D*—H⋯*A*	*D*—H	H⋯*A*	*D*⋯*A*	*D*—H⋯*A*
N2—H2*A*⋯F2^i^	0.86	1.99	2.849 (5)	174
N4—H4*A*⋯F1^i^	0.86	2.02	2.848 (4)	162
C1—H1⋯F2^ii^	0.93	2.46	3.317 (6)	153

Symmetry codes: (i) 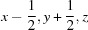; (ii) 
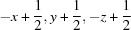
.

**Fig. 1.**
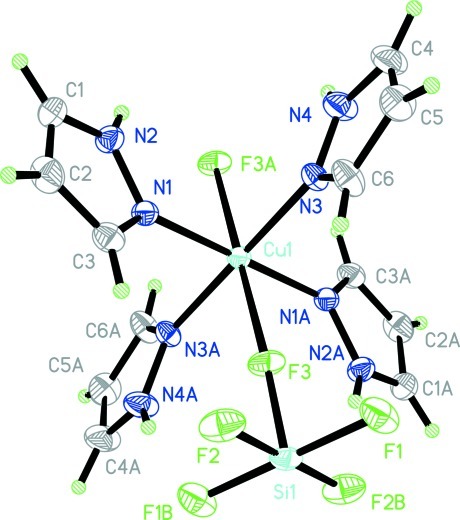
The molecular structure of (I). Displacement ellipsoids are drawn at the 30% probability level and H atoms are shown as small spheres of arbitrary radius.

**Fig. 2.**
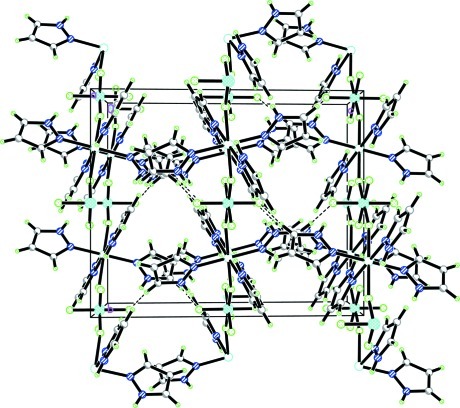
The crystal packing for (I).

## supplementary crystallographic information

### Comment

In recent years, nitrogen-containing ligands have been extensively studied (Li *et al.*, 2011). Pyrazole have been well used as a kind of N-containing ligands.

Single-crystal X-ray diffraction analysis reveals that the title compound (I) crystallizes in the monoclinic space group *C2/c*. For the title compound, the geometry of the Cu(II) ion is bound by four pyrazole ligands and two hexafluoridosilicate dianions, which illustrates a distorted octahedral coordination environment (Fig 1). The dihedral angle between the pyrazole rings is 105.6°.

### Experimental

A buffer layer of a solution (8 ml) of methanol and chloroform (1:1) was carefully layered over the chloroform solution of pyrazole (0.06 mmol, 6 ml). Then a methanol solution of CuSiF_6_ (0.02 mmol, 6 ml) was layered over the buffer layer. The resultant reaction was left to stand at room temperature. After *ca* three weeks, colorless blocks appeared at the boundary. Yield: ~20% (based on pyrazole).

### Refinement

C-bound H atoms were positioned geometrically and refined in the riding-model approximation, with C—H = 0.93 Å and *U*_iso_(H) = 1.2*U*_eq_ (C).

The N-bound H atoms were located in a difference map and their positions were freely refined with *U*_iso_(H) = 1.2*U*_eq_ (N).

### Crystal data

**Table:**

[Cu(SiF_6_)(C_3_H_4_N_2_)_4_]	*F*(000) = 964
*M**_r_* = 477.96	*D*_x_ = 1.692 Mg m^−^^3^
Monoclinic, *C*2/*c*	Mo *K*α radiation, λ = 0.71073 Å
Hall symbol: -C 2yc	Cell parameters from 7871 reflections
*a* = 10.617 (2) Å	θ = 3.0–27.5°
*b* = 12.108 (2) Å	µ = 1.30 mm^−^^1^
*c* = 14.652 (3) Å	*T* = 293 K
β = 95.07 (3)°	Block, colorless
*V* = 1876.2 (6) Å^3^	0.25 × 0.22 × 0.20 mm
*Z* = 4	

### Data collection

**Table:**

Rigaku Mercury CCD diffractometer	1665 independent reflections
Radiation source: fine-focus sealed tube	1283 reflections with *I* > 2σ(*I*)
Graphite monochromator	*R*_int_ = 0.079
Detector resolution: 9 pixels mm^-1^	θ_max_ = 25.0°, θ_min_ = 3.0°
ω scans	*h* = −12→12
Absorption correction: multi-scan (*CrystalClear*; Rigaku/MSC, 2005)	*k* = −14→14
*T*_min_ = 0.730, *T*_max_ = 0.771	*l* = −17→17
7864 measured reflections	

### Refinement

**Table:**

Refinement on *F*^2^	Primary atom site location: structure-invariant direct methods
Least-squares matrix: full	Secondary atom site location: difference Fourier map
*R*[*F*^2^ > 2σ(*F*^2^)] = 0.059	Hydrogen site location: inferred from neighbouring sites
*wR*(*F*^2^) = 0.101	H-atom parameters constrained
*S* = 1.16	*w* = 1/[σ^2^(*F*_o_^2^) + (0.0309*P*)^2^ + 3.0774*P*] where *P* = (*F*_o_^2^ + 2*F*_c_^2^)/3
1665 reflections	(Δ/σ)_max_ < 0.001
130 parameters	Δρ_max_ = 0.44 e Å^−^^3^
0 restraints	Δρ_min_ = −0.31 e Å^−^^3^

### Special details

**Table:**

Geometry. All e.s.d.'s (except the e.s.d. in the dihedral angle between two l.s. planes) are estimated using the full covariance matrix. The cell e.s.d.'s are taken into account individually in the estimation of e.s.d.'s in distances, angles and torsion angles; correlations between e.s.d.'s in cell parameters are only used when they are defined by crystal symmetry. An approximate (isotropic) treatment of cell e.s.d.'s is used for estimating e.s.d.'s involving l.s. planes.
Refinement. Refinement of *F*^2^ against ALL reflections. The weighted *R*-factor *wR* and goodness of fit *S* are based on *F*^2^, conventional *R*-factors *R* are based on *F*, with *F* set to zero for negative *F*^2^. The threshold expression of *F*^2^ > σ(*F*^2^) is used only for calculating *R*-factors(gt) *etc*. and is not relevant to the choice of reflections for refinement. *R*-factors based on *F*^2^ are statistically about twice as large as those based on *F*, and *R*- factors based on ALL data will be even larger.

### Fractional atomic coordinates and isotropic or equivalent isotropic displacement parameters (Å^2^)

**Table:**

	*x*	*y*	*z*	*U*_iso_*/*U*_eq_	
Cu1	0.2500	0.2500	0.5000	0.0262 (2)	
F1	0.3887 (2)	−0.0988 (2)	0.4884 (2)	0.0540 (8)	
F2	0.5087 (3)	0.0006 (2)	0.38606 (17)	0.0533 (8)	
F3	0.3866 (2)	0.09883 (19)	0.48968 (18)	0.0429 (7)	
Si1	0.5000	0.0000	0.5000	0.0313 (4)	
N1	0.2744 (3)	0.2900 (3)	0.3698 (2)	0.0304 (9)	
N2	0.2000 (3)	0.3633 (3)	0.3217 (3)	0.0362 (9)	
H2A	0.1452	0.4044	0.3451	0.043*	
N3	0.1011 (3)	0.1589 (3)	0.4510 (2)	0.0309 (9)	
N4	−0.0206 (4)	0.1865 (3)	0.4494 (3)	0.0490 (11)	
H4A	−0.0480	0.2459	0.4730	0.059*	
C1	0.2212 (5)	0.3646 (4)	0.2340 (3)	0.0476 (13)	
H1	0.1801	0.4084	0.1885	0.057*	
C2	0.3147 (5)	0.2895 (4)	0.2227 (3)	0.0487 (14)	
H2	0.3503	0.2723	0.1687	0.058*	
C3	0.3449 (4)	0.2448 (4)	0.3085 (3)	0.0389 (11)	
H3	0.4059	0.1907	0.3219	0.047*	
C4	−0.0944 (5)	0.1099 (4)	0.4063 (4)	0.0611 (17)	
H4	−0.1822	0.1118	0.3971	0.073*	
C5	−0.0190 (5)	0.0291 (4)	0.3785 (3)	0.0522 (15)	
H5	−0.0430	−0.0351	0.3467	0.063*	
C6	0.1020 (4)	0.0633 (4)	0.4078 (3)	0.0446 (13)	
H6	0.1749	0.0238	0.3984	0.054*	

### Atomic displacement parameters (Å^2^)

**Table:**

	*U*^11^	*U*^22^	*U*^33^	*U*^12^	*U*^13^	*U*^23^
Cu1	0.0254 (4)	0.0244 (4)	0.0285 (4)	0.0000 (4)	0.0012 (3)	−0.0008 (4)
F1	0.0361 (15)	0.0378 (16)	0.088 (2)	−0.0014 (13)	0.0064 (15)	−0.0160 (15)
F2	0.0553 (17)	0.0645 (19)	0.0392 (17)	0.0229 (16)	−0.0006 (14)	−0.0063 (15)
F3	0.0389 (15)	0.0348 (15)	0.0540 (18)	0.0193 (12)	−0.0006 (13)	−0.0025 (13)
Si1	0.0268 (9)	0.0263 (9)	0.0397 (11)	0.0087 (8)	−0.0032 (8)	−0.0072 (8)
N1	0.028 (2)	0.028 (2)	0.035 (2)	0.0005 (16)	−0.0005 (18)	0.0004 (17)
N2	0.035 (2)	0.038 (2)	0.036 (2)	0.0079 (18)	0.0058 (19)	0.0050 (19)
N3	0.026 (2)	0.028 (2)	0.039 (2)	−0.0013 (16)	0.0062 (17)	−0.0066 (17)
N4	0.034 (2)	0.031 (2)	0.080 (3)	0.0045 (19)	−0.005 (2)	−0.005 (2)
C1	0.042 (3)	0.064 (4)	0.036 (3)	−0.005 (3)	0.001 (2)	0.015 (3)
C2	0.047 (3)	0.069 (4)	0.032 (3)	−0.006 (3)	0.012 (2)	−0.007 (3)
C3	0.032 (2)	0.040 (3)	0.045 (3)	0.003 (2)	0.006 (2)	−0.004 (3)
C4	0.039 (3)	0.042 (3)	0.097 (5)	−0.009 (3)	−0.021 (3)	0.002 (3)
C5	0.062 (4)	0.041 (3)	0.052 (3)	−0.012 (3)	−0.009 (3)	−0.011 (3)
C6	0.038 (3)	0.041 (3)	0.057 (3)	−0.009 (2)	0.013 (3)	−0.018 (3)

### Geometric parameters (Å, º)

**Table:**

Cu1—N1^i^	2.007 (4)	N2—H2A	0.8600
Cu1—N1	2.007 (4)	N3—C6	1.320 (5)
Cu1—N3^i^	2.008 (3)	N3—N4	1.332 (5)
Cu1—N3	2.008 (3)	N4—C4	1.337 (6)
Cu1—F3^i^	2.348 (2)	N4—H4A	0.8600
Cu1—F3	2.348 (2)	C1—C2	1.366 (6)
Si1—F1	1.679 (2)	C1—H1	0.9300
Si1—F2	1.680 (3)	C2—C3	1.381 (6)
SI1—F3	1.695 (2)	C2—H2	0.9300
Si1—F1^ii^	1.679 (2)	C3—H3	0.9300
Si1—F2^ii^	1.680 (3)	C4—C5	1.350 (7)
Si1—F3^ii^	1.695 (2)	C4—H4	0.9300
N1—C3	1.336 (5)	C5—C6	1.381 (6)
N1—N2	1.345 (5)	C5—H5	0.9300
N2—C1	1.324 (6)	C6—H6	0.9300
			
N1^i^—Cu1—N1	180.0	C3—N1—N2	104.9 (4)
N1^i^—Cu1—N3^i^	87.50 (14)	C3—N1—Cu1	131.8 (3)
N1—Cu1—N3^i^	92.50 (14)	N2—N1—Cu1	122.6 (3)
N1^i^—Cu1—N3	92.50 (14)	C1—N2—N1	111.9 (4)
N1—Cu1—N3	87.50 (14)	C1—N2—H2A	124.0
N3^i^—Cu1—N3	180.00 (16)	N1—N2—H2A	124.0
N1^i^—Cu1—F3^i^	89.70 (12)	C6—N3—N4	105.1 (4)
N1—Cu1—F3^i^	90.30 (12)	C6—N3—Cu1	127.9 (3)
N3^i^—Cu1—F3^i^	91.15 (11)	N4—N3—Cu1	126.9 (3)
N3—Cu1—F3^i^	88.85 (11)	N3—N4—C4	111.2 (4)
N1^i^—Cu1—F3	90.30 (12)	N3—N4—H4A	124.4
N1—Cu1—F3	89.70 (12)	C4—N4—H4A	124.4
N3^i^—Cu1—F3	88.85 (11)	N2—C1—C2	107.3 (4)
N3—Cu1—F3	91.15 (11)	N2—C1—H1	126.3
F3^i^—Cu1—F3	180.0	C2—C1—H1	126.3
Si1—F3—Cu1	169.24 (15)	C1—C2—C3	105.2 (4)
F1^ii^—Si1—F1	180.00 (17)	C1—C2—H2	127.4
F1^ii^—Si1—F2^ii^	90.16 (15)	C3—C2—H2	127.4
F1—Si1—F2^ii^	89.84 (15)	N1—C3—C2	110.6 (4)
F1^ii^—Si1—F2	89.84 (15)	N1—C3—H3	124.7
F1—Si1—F2	90.16 (15)	C2—C3—H3	124.7
F2^ii^—Si1—F2	180.000 (1)	N4—C4—C5	107.8 (5)
F1^ii^—Si1—F3	89.69 (12)	N4—C4—H4	126.1
F1—Si1—F3	90.31 (12)	C5—C4—H4	126.1
F2^ii^—Si1—F3	89.49 (13)	C4—C5—C6	104.5 (4)
F2—Si1—F3	90.51 (13)	C4—C5—H5	127.8
F1^ii^—Si1—F3^ii^	90.31 (12)	C6—C5—H5	127.8
F1—Si1—F3^ii^	89.69 (12)	N3—C6—C5	111.4 (4)
F2^ii^—Si1—F3^ii^	90.51 (13)	N3—C6—H6	124.3
F2—Si1—F3^ii^	89.49 (13)	C5—C6—H6	124.3
F3—Si1—F3^ii^	180.0		
			
N1^i^—Cu1—F3—Si1	−53.9 (8)	N1—Cu1—N3—C6	73.5 (4)
N1—Cu1—F3—Si1	126.1 (8)	F3^i^—Cu1—N3—C6	163.9 (4)
N3^i^—Cu1—F3—Si1	33.6 (8)	F3—Cu1—N3—C6	−16.1 (4)
N3—Cu1—F3—Si1	−146.4 (8)	N1^i^—Cu1—N3—N4	78.8 (4)
Cu1—F3—Si1—F1^ii^	−48.7 (8)	N1—Cu1—N3—N4	−101.2 (4)
Cu1—F3—Si1—F1	131.3 (8)	F3^i^—Cu1—N3—N4	−10.9 (4)
Cu1—F3—Si1—F2^ii^	41.4 (8)	F3—Cu1—N3—N4	169.1 (4)
Cu1—F3—Si1—F2	−138.6 (8)	C6—N3—N4—C4	0.0 (5)
N3^i^—Cu1—N1—C3	87.4 (4)	Cu1—N3—N4—C4	175.7 (3)
N3—Cu1—N1—C3	−92.6 (4)	N1—N2—C1—C2	−0.5 (5)
F3^i^—Cu1—N1—C3	178.5 (4)	N2—C1—C2—C3	0.5 (6)
F3—Cu1—N1—C3	−1.5 (4)	N2—N1—C3—C2	0.0 (5)
N3^i^—Cu1—N1—N2	−104.4 (3)	Cu1—N1—C3—C2	169.7 (3)
N3—Cu1—N1—N2	75.6 (3)	C1—C2—C3—N1	−0.3 (6)
F3^i^—Cu1—N1—N2	−13.2 (3)	N3—N4—C4—C5	0.0 (6)
F3—Cu1—N1—N2	166.8 (3)	N4—C4—C5—C6	0.0 (6)
C3—N1—N2—C1	0.3 (5)	N4—N3—C6—C5	0.0 (5)
Cu1—N1—N2—C1	−170.6 (3)	Cu1—N3—C6—C5	−175.7 (3)
N1^i^—Cu1—N3—C6	−106.5 (4)	C4—C5—C6—N3	0.0 (6)

Symmetry codes: (i) −*x*+1/2, −*y*+1/2, −*z*+1; (ii) −*x*+1, −*y*, −*z*+1.

### Hydrogen-bond geometry (Å, º)

**Table:**

*D*—H···*A*	*D*—H	H···*A*	*D*···*A*	*D*—H···*A*
N2—H2*A*···F2^iii^	0.86	1.99	2.849 (5)	174
N4—H4*A*···F1^iii^	0.86	2.02	2.848 (4)	162
C1—H1···F2^iv^	0.93	2.46	3.317 (6)	153

Symmetry codes: (iii) *x*−1/2, *y*+1/2, *z*; (iv) −*x*+1/2, *y*+1/2, −*z*+1/2.
